# Integrated stress response plasticity governs normal cell adaptation to chronic stress via the PP2A-TFE3-ATF4 pathway

**DOI:** 10.21203/rs.3.rs-4013396/v1

**Published:** 2024-03-28

**Authors:** Rita A. Avelar, Riya Gupta, Gracie Carvette, Felipe da Veiga Leprevost, Jose Colina, Jessica Teitel, Alexey I. Nesvizhskii, Caitlin M. O’Connor, Maria Hatzoglou, Shirish Shenolikar, Peter Arvan, Goutham Narla, Analisa DiFeo

**Affiliations:** 1 Department of Pathology, The University of Michigan, Ann Arbor, MI 48109, USA.; 2 Rogel Cancer Center, The University of Michigan, Ann Arbor, MI 48109, USA.; 3 Department of Computational Medicine and Bioinformatics, University of Michigan, Ann Arbor, MI 48109, USA.; 4 Department of Internal Medicine, Division of Genetic Medicine, University of Michigan, Ann Arbor, MI 48109, USA.; 5 Department of Genetics and Genome Sciences, Case Western Reserve University, Cleveland, OH 44106, USA.; 6 Emeritus Professor, Duke-NUS Medical School, Singapore; 7 Professor Emeritus, Duke University School of Medicine, USA; 8 Division of Metabolism Endocrinology and Diabetes, University of Michigan Medical Center, Ann Arbor, MI 48109, USA.; 9 Department of Obstetrics and Gynecology, University of Michigan, Ann Arbor, MI 48109, USA.

## Abstract

The integrated stress response (ISR) regulates cell fate during conditions of stress by leveraging the cell’s capacity to endure sustainable and efficient adaptive stress responses. Protein phosphatase 2A (PP2A) activity modulation has been shown to be successful in achieving both therapeutic efficacy and safety across various cancer models; however, the molecular mechanisms driving its selective antitumor effects remain unclear. Here, we show for the first time that ISR plasticity relies on PP2A activation to regulate drug response and dictate cellular fate under conditions of chronic stress. We demonstrate that genetic and chemical modulation of the PP2A leads to chronic proteolytic stress and triggers an ISR to dictate cell fate. More specifically, we uncovered that the PP2A-TFE3-ATF4 pathway governs ISR cell plasticity during endoplasmic reticular and cellular stress independent of the unfolded protein response. We further show that normal cells reprogram their genetic signatures to undergo ISR-mediated adaptation and homeostatic recovery thereby successfully avoiding toxicity following PP2A-mediated stress. Conversely, oncogenic specific cytotoxicity induced by chemical modulation of PP2A is achieved by activating chronic and irreversible ISR in cancer cells. Our findings propose that a differential response to chemical modulation of PP2A is determined by intrinsic ISR plasticity, providing a novel biological vulnerability to selectively induce cancer cell death and improve targeted therapeutic efficacy.

## Introduction

Eukaryotic cells can adapt to various environmental challenges promptly and efficiently in order to promote cell survival. However, under chronic stress conditions, cells that cannot activate a sufficient adaptive response to mitigate stress and restore homeostasis are targeted for programmed cell death. One of the predominant signaling pathways known to regulate such adaptive response is the known as the integrated stress response (ISR), a sensor mechanism that promptly reacts to extrinsic and intrinsic stress factors including: 1) oxygen and amino-acid deprivation, 2) viral infection, 3) glucose imbalances, 4) oncogenic activation, and 5) overload/accumulation of misfolded proteins in the endoplasmic reticulum (ER) thereby triggering an unfolded protein response (UPR)^1–7^. When the ISR is unable to sustain stress adaptation mechanisms, anti-survival components become activated to execute cell death. ATF4 is the master regulator of ISR-mediated cellular adaptation during stress conditions, dictating the transcriptional activation of several genes involved in homeostatic recovery, stress response, amino acid biosynthesis, and CHOP-mediated autophagy and apoptotic signatures, ultimately dictating cell fate^5,8–10^. This decision is dictated by the nature, duration, space, and magnitude of both the stress and the induced ISR. Thus, ISR reversibility is an essential component of cell fate decision making, being instrumental for cell survival^11^. At basal states, cancer cells develop the unique ability to favor pro-survival phenotypes through the exploitation of ISR signaling, allowing them to withstand the high levels of protein synthesis driven by exacerbated oncogenic demand^12,13^. Aberrant responses to activated ISR signaling have been widely implicated in cellular transformation, cancer development, metastasis, and chemoresistance mechanisms in many human malignancies. In contrast, normal cells maintain greater basal adaptive plasticity, which enables them to recover from chronically induced ISR and restore their identity and primary function^14^.

Protein phosphatase 2A is a heterotrimeric serine/threonine tumor suppressive protein phosphatase that is comprised of three main components: PP2A-A – the scaffolding subunit, providing a nucleating platform through its conformational flexibility for heterotrimer formation –, PP2A-C – the catalytic subunit, which provides enzymatic activity directed against phosphorylated serine and threonine residues –, and PP2A-B – one of fifteen structurally distinct regulatory subunits that directs substrate specificity^15–17^. Given that cellular homeostasis is dysregulated in human cancers due to imbalances in protein phosphorylation, which results in global cellular signaling perturbations, the therapeutic targeting of phosphatases such as PP2A has emerged as an effective therapeutic strategy to reestablish cellular homeostasis^18–28^. DT-061 is a PP2A modulator that regulates this tumor suppressor’s activity by selectively binding to a unique pocket at the interface of the PP2A-Aα, Cα, and B56α subunits^29^. DT-061 demonstrates potent anticancer properties in numerous cancer models with high tolerability profiles, even in long-term *in vivo* dosing studies^21–28^. However, the role of DT-061 and other PP2A modulators in modulating human stress-mediated responses has yet to be elucidated, remaining unclear whether this could explain the promising safety profiles previously observed across numerous cancer models.

Despite the evident efficacy in targeted therapeutic strategies, a significant challenge resides in ensuring selective and specific oncogenic toxicity while sparing healthy tissues. The present study has sought to elucidate the mechanisms through which PP2A modulation specifically induces cell death in cancer cells. Herein, we demonstrated that DT-061 triggers ISR to dictate cell fate. Accordingly, under reversible and chronic stress conditions induced with DT-061 treatment, non-malignant human cells undergo transcriptional and translational reprogramming, leveraging their inherent ISR plasticity to survive and restore homeostasis, a mechanism previously observed to occur in normal tissues in order to overcome cellular stress^8^. This reprograming represses stress-mediated cell death, serving as an adaptive homeostatic mechanism that enables non-malignant tissues to restore basal functions, reinstate their cellular identity, and ultimately survive. Conversely, global proteomics and RNA-seq analyses revealed that PP2A modulation via DT-061 induces irreversible ISR in cancer cells, preventing them to resort to adaptive mechanisms that would enable cell survival. Therefore, whereas DT-061 elicits ISR downstream pathways associated with chronic stress and cell death responses in cancer cells, it simultaneously activates distinct ISR-mediated signatures that confer adaptive and protective mechanisms in non-transformed cells, contributing to their survival. Mechanistically, we uncovered that DT-061-mediated modulation of PP2A induced the ISR through the dephosphorylation of TFE3, resulting in chronic upregulation of ATF4 and CHOP to inhibit stress recovery and induce cell death in cancer cells. Importantly, our current studies provide evidence that the PP2A/TFE3 axis is a molecular process essential to dictate ISR plasticity, supporting a promising avenue for cancer-selective therapies based on manipulating PP2A-mediated irreversible ISR, while also providing novel insight into DT-061’s anti-tumor properties.

## Results

### Stress response and cell death pathways are enriched in cancer cells treated with DT-061

Recently, our group demonstrated that a large majority of high-grade serous cancer (HGSC) tumors have loss of several PP2A genes that are essential for driving cellular transformation, which correlates with poor patient survival^21^. We also found that the PP2A modulator, DT-061, was able to stabilize and regulate the remnant copy of PP2A-Aα, biasing specific PP2A heterotrimeric pools to drive its tumor suppressive function, both as monotherapy as well as in combination with PARP inhibitors in numerous patient-derived xenograft models^21^. Interestingly, despite the potent cytotoxic effects DT-061 produces in malignant tissues, normal cells are able to adapt and circumvent pro-death signals, resulting in a large therapeutic index and high tolerability profiles *in vivo*^21–28^. Herein, our studies seek to investigate the fundamental mechanism responsible for the malignant specificity displayed by this class of drugs and further investigate the molecular mechanism that dictates that selectivity.

**Figure 1 –:**
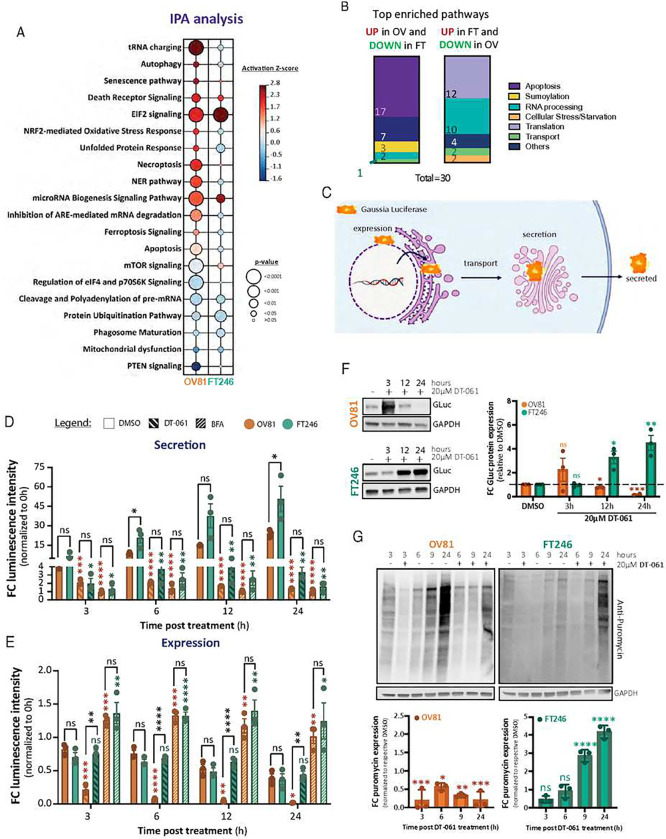
Stress response, Protein and RNA metabolism, cellular transport, and pro-death pathways are significantly enriched in DT-061-treated cells. A) Ingenuity Pathway Analysis (IPA) identifies ER stress-related and cellular adaptation pathways such as t-RNA charging, EIF2, NRF2-mediated oxidative stress response, and UPR, as well as cell death including autophagy, senescence, death receptor, and necroptosis signaling, cascades among the topmost significantly induced pathways in HGSC cancer cells after 3 hours of DT-061 relative to DMSO. The color scale represents Z-score activation and circles represent the statistical significance. B) Top enriched pathways categorized by overall functional relevance and pathway clustering. Data separated by upregulated in OV and simultaneously downregulated in FT (left) or vice-versa (right). C) Schematic representing the expression and secretion cycle of Gaussia luciferase, depicting each important step for its efficient detection. This system was utilized as a reporter assay to monitor the secretory pathway and measure ER stress as previously described by Badr *et al.*^31^. D) Fold change luminescence intensity relative to 0h DMSO. Media was collected at 3, 6, 12, and 24 hours after treatment with DMSO, DT-061, or BFA as an indirect measurement of secretion ability. Data presented as the mean ± SD (n=3), (one-way ANOVA with multiple comparisons, comparing the mean of each column with the mean of DMSO control column, ns > 0.05, *p < 0.05, **p < 0.01, ***p < 0.001, ****p < 0.0001). E) Fold change luminescence intensity relative to 0h DMSO. Cell lysates were collected at 3, 6, 12, and 24 hours after treatment with DMSO, DT-061, or BFA as an indirect measurement of protein expression. Data presented as the mean ± SD (n=3), (one-way ANOVA with multiple comparisons, comparing the mean of each column with the mean of DMSO control column, ns > 0.05, *p < 0.05, **p < 0.01, ***p < 0.001, ****p < 0.0001). F) Protein analysis of Gaussia luciferase expression was performed via western blotting (left), with its respective quantification represented (right). G) Puromycin 10-minute pulse with 50-minute chase labeling experiments in the presence of DMSO or DT-061 after 3, 6, 9, and 24 hours incubation, to measure newly translated protein. Western blotting analysis was used to quantify puromycin incorporation and GAPDH as the loading control (top). Whole lane quantification was graphed as fold change in puromycin expression relative to respective DMSO over time (bottom). Data presented as the mean ± SD (n=3), (one-way ANOVA with multiple comparisons, comparing the mean of each column with the mean of 3h DMSO control column (not represented) for respective cell line, ns > 0.05, *p < 0.05, **p < 0.01, ***p < 0.001, ****p < 0.0001).

To identify response differences and distinct translational signatures in DT-061 treated cancer versus non-transformed cells, we first pursued an unbiased global proteomics approach and compared a patient-derived HGSC cell line (OV81) to non-malignant fallopian tube cells (FT246) treated with DMSO or DT-061 for 3 hours. Ingenuity Pathway Analysis (IPA) identified t-RNA charging, EIF2, NRF2-mediated oxidative stress response, UPR, and pre-RNA modulation signaling cascades among the most significantly induced pathways in HGSC cancer cells after DT-061 exposure relative to DMSO (Fig. 1A). Indeed, all IPA annotated pathways, involving stress responses and cell death signaling, including autophagy, senescence, death receptor, necroptosis, ferroptosis, and apoptosis, were significantly induced by DT-061 treatment in OV81 cells compared to FT246 cells. Interestingly, while several stress response-related pathways such as t-RNA charging, NRF2-mediated oxidative stress response, and UPR were exclusively upregulated in the cancer cells after DT-061 exposure, the Death Receptor and EIF2 signaling pathways were highly activated in both the malignant and non-transformed contexts. Such results imply that both cell lines respond to PP2A modulation by activating cellular stress responses, although distinct downstream signatures that regulate cell fate are observed.

To further understand the distinct molecular mechanisms that malignant vs. non-transformed cells utilize to respond to the DT-061-induced cellular stress, we then evaluated the protein networks perturbed with opposing trends in the OV81 and FT246 lines. Reactome Pathway Enrichment (RPE) analysis revealed that in the presence of DT-061, apoptosis and SUMOylation were the two pathways most significantly upregulated in OV81 but downregulated in FT246 (Fig. 1B and ***Supplemental Fig. 1A-B***). On the other hand, translation, RNA processing, and protein transport were significantly enriched in FT246 cell line while the opposite trend was noted in the OV81 cell line. This further supports our IPA analysis, as it identified mediators of cap-dependent translation and master regulators of ISR and stress cellular responses as the most significantly upregulated pathways in the DT-061 treated OV81 cell line. To validate our findings and to potentially generalize the observations to other cancer contexts, we performed an RNA-seq analysis using H358, a non-small cell lung cancer cell line sensitive to DT-061 treatment with global and phospho-proteomics analyses previously established^30^. Consistent with our current proteomics findings in the OV81 cell line, DT-061 treatment significantly upregulated genes associated with ER stress in H358 cells, including HERPUD1, ATF3, DDIT3 (CHOP), TRIB3, PPP1R15A (GADD34), along with other effectors (***Supplemental Fig. 1C***). Further RPE and STRING analyses of these candidates included the UPR with IRE1a and ATF6 chaperone activation as well as EIF2, PERK, and ATF4 activated genes (***Supplemental Fig. 1D*** and ***Supplemental Fig. 1E***). These molecules are all well-recognized components of the integrated stress response (ISR) pathway.

As previously published by Wiredja *et al*.^30^ and identified in our current proteomics study, DT-061 treatment can impair ER to Golgi transport, which potentially leads to accumulation of proteins in the ER lumen that might explain UPR induction in PP2A modulator treated cell lines. To confirm this, we utilized a secretory form of Gaussia luciferase^31^ to monitor secretory efficiency in our cancer and non-malignant cells (Fig. 1C). As a positive control we evaluated changes in protein secretion upon treatment with brefeldin A (BFA), a potent ER-to-Golgi transport inhibitor. As expected, in both cell lines BFA significantly reduced the ability to secrete luciferase, while protein expression remained unchanged. In contrast, DT-061 disrupted luciferase secretion to the same extent as BFA in both OV and FT lines (Fig. 1D) but selectively reduced the protein expression in OV81 cells as early as 3 hours post-treatment (Fig. 1E). Upon treatment with DT-061, both OV81 and FT246 cells presented with an increase in Gaussia luciferase mRNA expression; however, only OV81 cells exhibited a significant decrease in Gaussia luciferase protein levels (Fig. 1F and ***Supplemental Fig. 1F***).

Combining these findings with the discovery that p-eIF2α, a master regulator of protein translation, is highly induced by DT-061, we sought to determine the effect of PP2A modulation on general mRNA translation. To do so, we performed pulse-chase puromycin labeling utilizing the SUrface SEnsing of Translation (SUnSET) assay in OV81 and FT246 cell lines in order to measure nascent polypeptide chain formation (Fig. 1G). By 6 hours of DT-061 treatment OV81 cells exhibited a marked attenuation of the ability to translate protein whereas FT246 presented a translational recovery (Fig. 1G). Thus, chronic prolonged stress induced by DT-061 combined with an inability to restore translational cues may lead to cell death in cancer cells (Fig. 1G), while normal cells successfully activate adaptive pro-survival mechanisms, including RNA processing, protein translation, and anti-apoptotic cues.

Collectively, our proteomics and RNA-seq findings complemented by protein trafficking studies indicate that DT-061 treatment enables FT246 cells to partially recover from stress-induced translation, transcription, and transport inhibition, facilitating their survival. Conversely, OV81 cancer cells exhibit compromised translation capabilities along with activated pro-apoptotic stress responses, stemming from their incapacity to initiate parallel adaptive mechanisms, ultimately resulting in cell death.

### DT-061 activates ISR signaling in HGSC and FT cells to dictate cell fate

**Figure 2 –:**
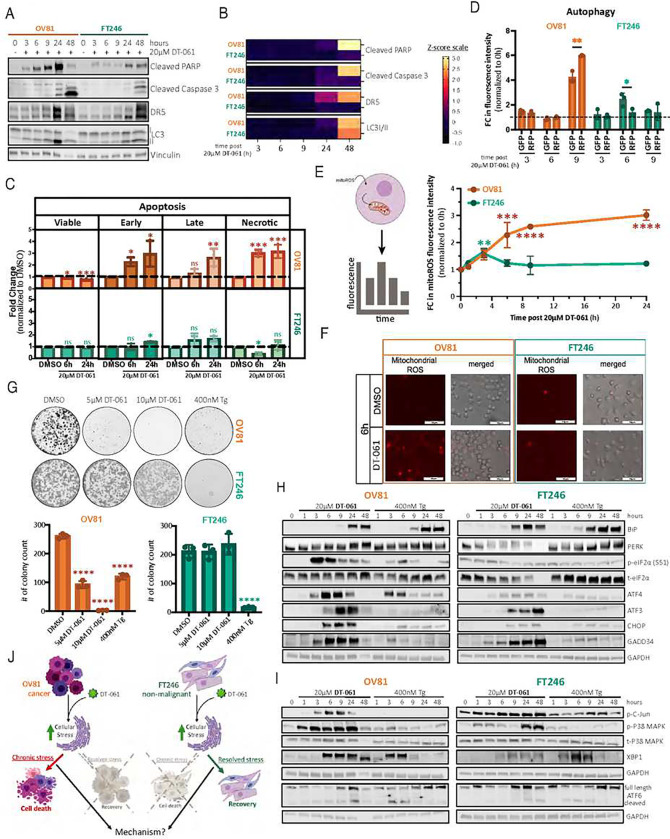
DT-061-mediated chronic and irreversible activation of the ISR stress dictates cell fate. A) Western blotting analysis evaluating the expression of cell death, apoptosis, and autophagy markers (cleaved PARP, cleaved Caspase 3 and DR5, and LC3I/II, respectively) upon DT-061 treatment. B) Representation by heatmap of Z-score values obtained after western blot quantification using image J and normalization to Vinculin as the loading control (from panel A). C) Annexin V and PI staining quantifying viable cells (Annexin V− and PI−), in early (Annexin V+ and PI−), late (Annexin V+ and PI+) or necrotic (Annexin V− and PI+) states after DT-061 treatment for 6 and 24 hours. Results for OV81 are graphed in orange while FT246 in green. Data presented as the mean ± SD (n=3), (one-way ANOVA with multiple comparisons, comparing the mean of each column with the mean of DMSO control column for its respective time point and cell line, ns > 0.05, *p < 0.05, ***p < 0.001). D) Premo™ Autophagy Tandem Sensor RFP-GFP-LC3B was used to detect the expression of LC3II post 3, 6, and 9 hours of DT-061 exposure. RFP expression correlates with late maturation and pH resistant vesicles (=autolysosomes) while GFP represents premature phagosomes sensitive to pH changes (=autophagosomes). Data presented as the mean ± SD (n=3), (unpaired Student T-tests, comparing RFP expression relative to GFP for each individual time point and respective cell line, *p < 0.05, **p < 0.01). E) Schematic explaining the experimental flow using mitoROS reagent to detect Reactive Oxygen Species (ROS) accumulation in cells after DT-061 exposure over time (1, 3, 6, 9, and 24 hours) (top). Obtained results were graphed measuring the fold change of fluorescence intensity under DT-061 conditions normalized to its respective DMSO control for each time point in OV81 (orange) and FT246 (green) (bottom). Data presented as the mean ± SD (n=3), (unpaired Student T-tests, comparing DT-061 treatment relative to 0h time point, **p < 0.01, ***p < 0.001, ****p < 0.0001). F) Immunofluorescent microscopy figures representative of the 6 hour time point quantified in (E). Red signal – mitoROS dye incorporated by mitochondria that are positive for ROS activity. Merged – red fluorescent signal and bright field pictures of the same area overlapped. G) Representative images of the clonogenic assay testing OV81 and FT246 cells’ ability to form colonies in the presence of DMSO, 5μM of DT-061, 10μM of DT-061, or 400nM of Tg for two weeks (left). Quantification is represented (right) and calculated as the mean ± SD (n=3), (one-way ANOVA with multiple comparisons, comparing the mean of each column with the mean of DMSO control for its respective cell line, ****p < 0.0001). H) and I) Western blotting analysis assessing DT-061-mediated activation of every arm of the UPR in comparison to Thapsigargine (Tg) (positive control). H) PERK, and I) IRE1a and ATF6 pathways and respective downstream targets were thoroughly analyzed under different exposure times to DT-061 or Tg in cancer (OV81) and non-malignant (FT246) cellular models. J) Schematic representing DT-061’s impact in cancer versus normal cellular fate. Our results show that upon DT-061 exposure, both cancer and non-malignant cells trigger stress signals, requiring adequate ER stress response mechanisms to restore homeostasis. OV81 cells are unable to respond to such cues efficiently and in a timely manner, activating chronic stress pathways, which result in cell death. On the other hand, normal cells are able to completely avert chronic and irreversible ER stress induction by activating efficient pro-survival feedback mechanisms, counteracting the activation of ER stress response pathways that allow for full recovery and cell survival.

The ER stress response includes ATF6 and IRE1α signaling along with the PERK pathway that is one of four kinases initiating the ISR, working closely to interact in a complex interconnected network to regulate cell fate. Together, these signaling pathways, predominantly PERK, can lead to the accumulation of CHOP and other cell fate-dictating proteins, eventually triggering apoptosis, stress-mediated autophagy, and ROS accumulation signals that will lead to cell death (***Supplemental Fig. 2A***). Our next step focused on identifying which signaling pathways were activated by DT-061 in HGSC lines and whether they were exclusive to cancer cells. We found that all markers of apoptosis (cleaved PARP, cleaved Caspase 3), stress mediated cell death (DR5), and autophagy, (LC3I/II) were significantly induced after DT-061 treatment in both HGSC and FT cells over time, although with a significantly greater intensity and more rapid kinetics in OV81 cancer cells (Fig. 2A, Fig. 2B and ***Supplemental Fig. 2B***). Studies of Annexin V-PI after 6 and 24 hours of DT-061 treatment revealed a significant increase in Annexin V levels, which correlated with the upregulation of apoptotic protein markers in OV81 cells, leading to a ~35% decrease in cell viability after 24 hours (Fig. 2C). Conversely, although early apoptosis is starting to peak at 24 hours in FT246 cells, no significant apoptotic-triggered cell death was detected at earlier time points post DT-061 exposure, corroborating our western blot results showing cleaved Caspase 3 being induced only after 48 hours of continuous 20μM of DT-061 treatment (Fig. 2A and Fig. 2B).

Differences in the kinetics and magnitude of autophagosome and autolysosome formation in DT-061 treated cells were detected by fluorescence intensity analysis of the autophagy associated protein LC3B. We observed that OV81 only forms autophagosomes after 6 hours of DT-061 exposure, although undergoing quick maturation after 9 hours of treatment with a 6-fold increase in mature autolysosomes (Fig. 2D). However, FT246 cells present with a 2-fold induction in autophagosome formation at 6 hours of DT-061 treatment, which is then reversed and reset to baseline levels after 9 hours of drug exposure. Furthermore, we found that, whereas both cell lines show a 1.5-fold increase in ROS accumulation after 3 hours of continuous DT-061 exposure, FT246 cells have the unique ability to restore ROS to baseline levels after 6 hours of treatment (Fig. 2E and Fig. 2F). OV81 cells were unable to reinstate ROS levels back to baseline, reaching a 3-fold increase in its accumulation after 24 hours of continuous treatment. We then compared the effects of DT-061 to those of thapsigargin (Tg), a potent ER stress/UPR inducer via the inhibition of the sarco/endoplasmic Ca^2+^ ATPase (SERCA) pump^32^. As previously observed^21^, DT-061 efficiently abrogated the ability of OV81 to form colonies, which we herein confirmed using 5μM and 10μM DT-061 concentrations and further compared to 400nM of Tg (Fig. 2G). Despite Tg triggering similar robust cytotoxic effects in both FT246 and OV81, FT246 colonies were able to grow under 5μM and 10μM of DT-061 exposure after two weeks of treatment. Importantly, OV81 and FT246 presented with widely different response patterns to short-term 20μM of DT-061 exposure (<24 hours) (***Supplemental Fig. 2C***) but very similar cell-death phenotypes in response to short and long-term Tg treatment, including the expression profiles of PARP cleavage, caspase 3 activation, and induction of DR5 and LC3II (***Supplemental Fig. 2D*** and ***Supplemental Fig. 2E***). These data suggest that DT-061 differentially activates pro-death stress responses that are distinct from the common stress responses shared by FT246 and OV81 cells upon challenge with a classical ER stressor.

To understand these differences, we proceeded to investigate the effects of DT-061 on the downstream proteins on all arms of the UPR. As expected, in both OV81 and FT246 cells, Tg was able to fully activate the PERK, IRE1α, and ATF6 pathways, and their downstream targets, including ATF4, ATF3, CHOP and GADD34 and p-P38 and XBP1, eventually returning to baseline levels (~9 hours), which would suggest stress adaptation (Fig. 2H and 2I, ***Supplemental Fig. 2F–2H***). Upon DT-061 treatment, PERK hyperphosphorylation appeared activated as early as 1 hour, leading to increased eIF2α (Serine-51) phosphorylation and subsequent expression of ATF4, ATF3, CHOP, BiP, and GADD34 proteins (Fig. 2H). The expression of GADD34 at 3 hours of DT-061 treatment can account for the subsequent decrease in p-eIF2α-S51 after 6 hours of exposure to DT-061. The PERK pathway induction in DT-061-treated FT246 cells – including PERK itself in addition to p-eIF2a-S51, ATF4, ATF3 – all appeared significantly attenuated in their responses in comparison to OV81 cells (Fig. 2H), which also includes CHOP, a well-known transcriptional activator of cell death. Moreover, DT-061 treatment was also able to activate the IRE1α pathway via increased p-C-Jun, p-P38, and XBP1 expression, which was greater in OV81 than in FT246 cells (Fig. 2I). Overall, DT-061 induces the expression of downstream proteins associated with all branches of the UPR in both non-malignant and cancer cells, with the effects consistently more pronounced in the HGSC cells.

### Chronic stress induced by DT-061 is irreversible and determines the fate of HGSC cells

**Figure 3 –:**
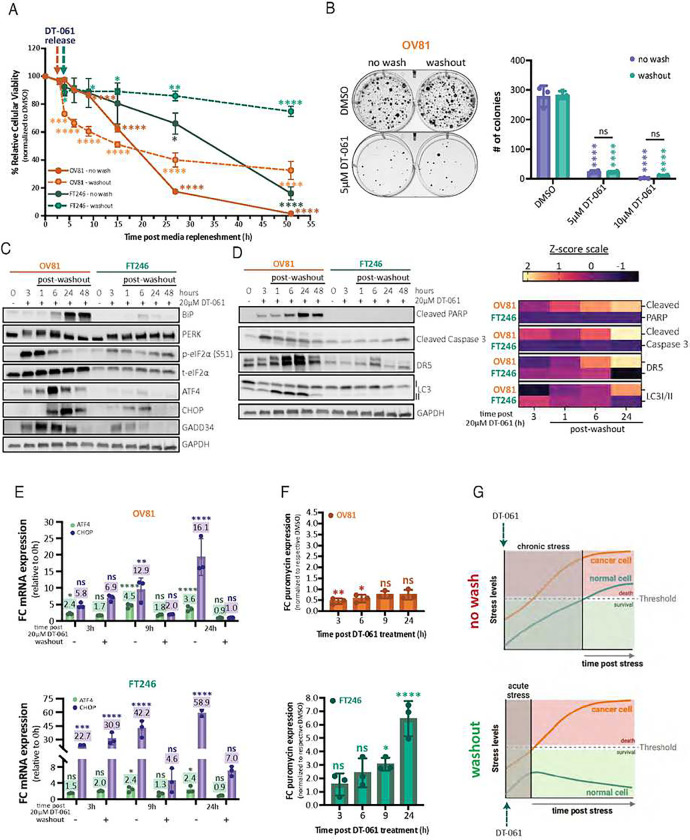
DT-061 induces chronic ISR activation, triggering cell death signaling pathways that are rescuable in normal cells but irreversible in HGSC. A) OV81 and FT246 cells were pre-incubated with DMSO or 20μM of DT-061 for 3 hours, followed by continuous treatment (nowash) or replenished with fresh media lacking drug (washout). Cellular viability was measured over time using cell titer glo and the cell viability percentage was calculated relative to 0h. Data presented as the mean ± SD (n=3) (one-way ANOVA with multiple comparisons, comparing the mean of each data point with the mean of 0 hour time point (100%) for its respective cell line, *p < 0.05, **p < 0.01, ****p < 0.0001). B) OV81 clonogenic assays evaluating the effect of DMSO versus 5μM and 10μM of DT-061 treatment in no-wash and washout conditions. No-wash conditions were replenished with fresh drug media every 3 days whereas washout cells were treated with drug once for 3 consecutive days followed by fresh drug-free media every three days until the end of the experiment (top). Quantification is represented (bottom) and calculated as the mean ± SD (n=3), (one-way ANOVA with multiple comparisons, comparing the mean of each column with the mean of their own respective DMSO control column, ****p < 0.0001). C) and D) Western blotting analysis evaluating the molecular mechanism and profiles of DT-061 post washout versus no-wash. The PERK pathway and its downstream targets C) as well as the ER stress-mediated cell death and autophagy markers D) were assessed in detail. Representation by heatmap of Z-score values was obtained after western blot quantification using image J and normalization to housekeeping (bottom) for each cell line and time point. Calculated z-scores were obtained from protein quantification of three independent biological replicates. E) mRNA from washout and no-wash experiments were extracted and qPCR analysis of ATF4 and CHOP expression levels was performed for OV81 (top) and FT246 (bottom). Data presented as the mean ± SD (n=3) (one-way ANOVA with multiple comparisons, comparing the mean of each column with the mean of DMSO control column for its respective target and cell line, ns > 0.05, *p < 0.05, **p < 0.01, ****p < 0.0001). F) Graphs representing the fold change in puromycin signal relative to DMSO for OV81 (top) and FT246 (bottom), with 10-minute pulse and 50-minute chase puromycin labeling for the washout protein lysates collected under the same conditions as C) and D). Data presented as the mean ± SD (n=3), (one-way ANOVA with multiple comparisons, comparing the mean of each column with the mean of 3h DMSO control column (not represented) for its own respective cell line, ns > 0.05, *p < 0.05, **p < 0.01, ****p <0.0001). G) Schematic demonstrating the differences in cancer and normal cells responses to chronic (top) versus acute (bottom) stress mediated by DT-061. If stress is chronically induced by constant exposure to DT-061, both malignant and non-malignant cells will be targeted for cell death. However, as cancer cells have a higher yield of baseline protein expression to sustain oncogenic signaling, thus leading to lower thresholds to homeostatic imbalances and inherently higher baseline stress levels, DT-061-mediated acute stress will still lead to death. Oppositely, normal cells are capable of rapidly adapting and promptly responding to DT-061-mediated ER stress cues under acute conditions, inducing pro-survival signals that eventually restore homeostasis. (Biorender was utilized to design schematics on panel G)).

So far, our data suggests that the ISR is a crucial component of the cellular response to stress induced by DT-061. Particularly, cancer cells exhibit a limited capacity in initiating adaptive mechanisms that restore cellular homeostasis, resulting in pro-death signals to dominate upon DT-061 exposure. In contrast, non-transformed cells are able to engage adaptive homeostatic mechanisms, having the inherent ability to recover from stressful occurrences by selectively activating specific feedback mechanisms that regulate cellular fate, ultimately surviving DT-061-induced ISR (Fig. 2J). To further investigate the differences in stress response that may trigger death selectively in cancer cells upon DT-061 treatment, we proceeded with drug washout studies. After evaluating cell viability profiles, we observed that continuous (no wash) DT-061 treatment in both OV81 and FT246 eventually led to around 100% cell death by 48 hours (100% and 85%, respectively) (Fig. 3A). However, although OV81 cells were unable to fully recover after washout conditions (going from 100% to ~80% of cell death and remaining at 92% of reduced colony formation, in Fig. 3A and Fig. 3B, respectively), release of DT-061 pre-incubation led to a greater recovery (~60%) of viability in FT246 cells (going from 16% (no wash) to 75% (washout) cell viability) (Fig. 3A). To better understand which molecular signatures could explain such phenotypic differences, we collected DT-061-treated cells after washout and no wash conditions to evaluate protein (Fig. 3C and Fig. 3D) and RNA expression changes (Fig. 3E). Strikingly, despite the PERK phosphorylation attenuation at 24 hours post-washout, all targets canonically downstream of eIF2α phosphorylation including ATF4, CHOP, and GADD34 remained activated in OV81 cells (compared to no-wash), at both the protein (Fig. 3C and ***Supplemental Fig. 2I***) and mRNA levels (Fig. 3E). The sustained suppression of these protein targets following drug washout suggests that OV81 cells are inherently incapable of rapidly restoring homeostasis prior to the accumulation of pro-death signals, essentially dictating their irreversible cell fate, which culminates in cell death despite DT-061 removal (Fig. 3D). In contrast, FT246 cells showed reversibility of stress signaling, with significant recovery of ATF4, CHOP, GADD34, and BiP protein expression. Interestingly, in OV81, ATF4 and CHOP mRNA expression are significantly induced after DT-061 treatment and only fully rescued to baseline levels after 24 hours of drug washout (Fig. 3E *– upper panel*). In FT246 cells, although ATF4 returns to baseline levels 9 hours post DT-061 washout (Fig. 3E *– lower panel*), CHOP mRNA expression remains significantly elevated, attaining higher levels than OV81 cells, persisting even after DT-061 removal (Fig. 3E *– upper panel*). Consistent with our no-wash results, nascent protein translation analysis post DT-061 washout revealed that FT246 still sustains stress-induced translation levels after drug removal, while translation in OV81 cells remains inhibited (Fig. 3F *– lower panel*). In the presence of DT-061, FT246 promptly reactivates the transcriptional machinery to combat reinstated stress, whereas cancer cells are inherently susceptible to apoptosis. Despite elevated CHOP and ATF4 mRNA, FT246’s translational machinery selectively limits ISR gene production by enhancing overall protein translation, a mechanism not observed in OV81. These molecular phenotypes extend to other ovarian cancer and non-malignant models (***Supplemental Fig. 2J*** and ***Supplemental Fig. 2K***), including FT237 and various HGSC cancer cell lines, corroborating ISR activation and cell fate disparities upon DT-061 treatment.

Collectively, the foregoing data suggest that prolonged exposure to DT-061 can ultimately result in both malignant and non-transformed cells sharing the same outcome: cell death (Fig. 3G *– upper panel*). Nevertheless, unlike cancer cells, normal cells can quickly adapt to acute stressors and dynamic environments, triggering pro-survival signals that facilitate the activation of adaptive rheostatic mechanisms owing to their increased basal ISR plasticity (Fig. 3G *– lower panel*).

### PP2A-mediated regulation of the ATF4-CHOP pathway via DT-061 is independent of the UPR

**Figure 4 –:**
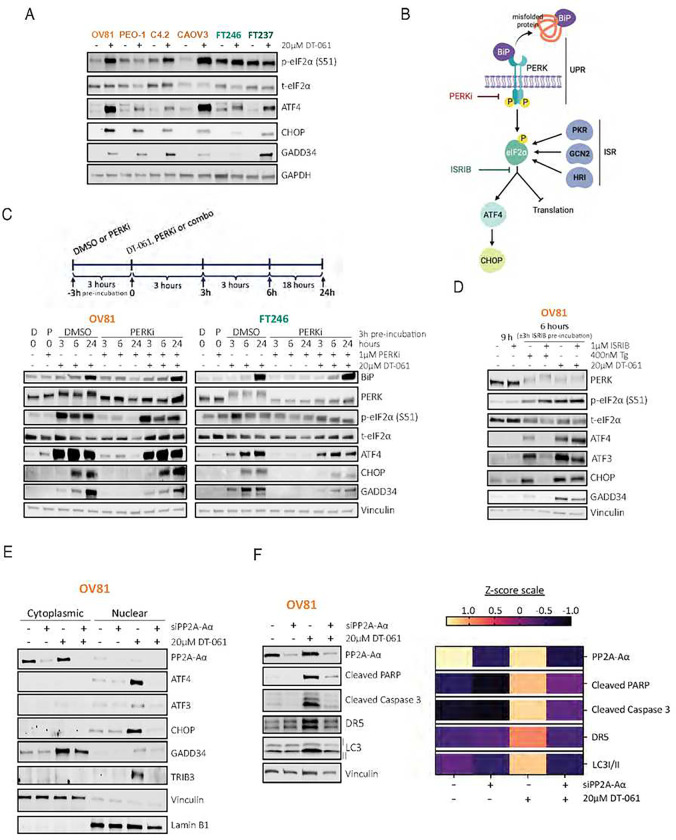
DT-061-mediated ATF4-CHOP activation is regulated independently of PERK activity and eIF2α phosphorylation. A) Western blotting analysis evaluating the effect of DT-061 treatment on eIF2α phosphorylation as well as ATF4 and CHOP expression in multiple HGSC and FT lines to identify dependency profiles. OV81, PEO-1, PEO-C4.2, and CAOV3 represent HGSC models (shades of orange) while FT246 and FT237 represent non-malignant fallopian tube tissues (shades of green). B) Schematic representing the two well-established canonical pathways that phosphorylate eIF2α resulting in ATF4 and CHOP increased expression: PERK (via the UPR) and PKR, GCN2, and HR (as part of the Integrated Stress Response (ISR)). Chemical inhibitors utilized to test PERK and eIF2α contribution to DT-061-mediated ATF4 and CHOP activation are also represented. C) Western blot analysis assessing the role of PERK activation and subsequent UPR regulation in OV81 and FT246 cells in the presence of DT-061. Cells were pre-incubated with either DMSO or 1μM of PERK inhibitor (PERKi) for 3 hours. Subsequently, DT-061 (DMSO pre-incubated), fresh PERKi, or the combination of the two drugs (PERKi pre-incubated) were added and cells were harvested after 3, 6, and 24 hours of the second round of drug exposure. PERK pathway activity was measured by assessing its hyperphosphorylation state (band shift) and downstream targets’ expression. D) Western blot analysis assessing the role of p-eIF2α downstream signaling and its impact in the activation of ATF4 and CHOP proteins in OV81 cells after treatment with DT-061. Cells were pre-incubated with either DMSO or 1μM of ISR Inhibitor (ISRIB) for 3 hours. Cells were then treated with either Tg or DT-061 after both DMSO and ISRIB pre-incubation and harvested at 6 hours post-second treatment round (total of 9 hours). Expression of ATF4 and its downstream targets were evaluated under each of these treatment conditions and quantified (in ***Suppl. Fig. 2K***). E) A pool of siRNAs was used to knockdown PP2A-Aα protein expression for 24 hours. Western blotting analysis was used to assess cytoplasmic versus nuclear localization as well as expression of the ATF4 and its downstream target genes after DT-061 treatment in both siControl (negative control) and siPP2A-Aα. F) Whole cell lysate of the conditions previously described in E) was also collected to evaluate the expression of cell death markers and ER-associated autophagy. Western blotting analysis was performed (left) and represented as z-score values in a heatmap obtained through western blot quantification (right). Calculated z-scores were obtained from protein quantification of three independent biological replicates.

Given the persistent activation of ATF4 and CHOP even after DT-061 washout in OV81 cells, we endeavored to gain deeper insights into their regulatory mechanisms. Although all HGSC and FT lines tested showed ATF4 and CHOP protein upregulation with DT-061 treatment (Fig. 4A), p-eIF2α expression was not consistent across all different ovarian lines. Thus, we wanted to evaluate whether the activation of these gene products might alternately depend upon PERK, other genes that control p-eIF2α levels, or pathways independent of p-eIF2α. Notably, in cells pre-treated with the PERK inhibitor (PERKi, Fig. 4B) prior to DT-061 exposure, PERK activation was effectively repressed (as observed by the reduced gel shift, ie., suppressed hyperphosphorylation). However, phosphorylation of eIF2α persisted, which was accompanied by increased expression of ATF4, CHOP, and GADD34, to the same extent as DT-061 alone (Fig. 4C). This suggests ISR activation by DT-061 that is independent of PERK activity. Lastly, PERKi co-treatment with DT-061 yields similar effects on cellular viability as DT-061 alone, showing PERK inhibition to have no impact on cancer cells to prevent a stress response (***Supplemental Fig. 2L*** – *upper panel*). Conversely, as non-malignant cells have an increased basal state of stress response due to higher inherent levels of p-eIF2α expression, FT246 cells have reduced viability over time with PERKi alone or in combination with DT-061 (***Supplemental Fig. 2L*** – *lower panel*).

To then test whether p-eIF2α activity is required for the DT-061-mediated ATF4, CHOP, and cell death, we used the general ISR Inhibitor (ISRIB), a molecule that inhibits the bioactivity of p-eIF2α to suppress ISR signaling^33^. As a positive control, ISRIB pre-incubation rescued the expression of ATF4, ATF3, CHOP, GADD34, and TRIB3 proteins induced by Tg alone^34^ (Fig. 4D and ***Supplemental Fig. 2M***). However, ISRIB pre-treatment could not block the notable induction of ATF4, ATF3, CHOP, GADD34, and TRIB3 proteins in response to DT-061 treatment in OV81 cells. These findings imply that DT-061 triggers the ATF4-CHOP pathway independently of PERK activation and eIF2α phosphorylation, possibly involving an alternative mechanism that regulates the observed ISR molecular signatures. Given this surprising outcome, we then sought to confirm that DT-061-mediated induction of ATF4 was modulated by PP2A activity. DT-061 significantly activated ATF4, ATF3, CHOP, and TRIB3, guaranteeing their localization into the nucleus, where they can act as transcription factors and propagate autophagy, apoptotic, and necrotic ER-associated signals (Fig. 4E). However, PP2A inhibition by siRNA-mediated knockdown of PP2A-Aα, an essential component of PP2A holoenzyme activity, significantly abrogated the ability of DT-061 to activate or translocate these targets into the nucleus, suggesting that such molecular events do require PP2A (Fig. 4E). Cell death and autophagy markers were also found to be significantly rescued in PP2A-Aα knockdown after 6 hours of treatment with DT-061 (Fig. 4F).

Together these data suggest that DT-061-mediated activation of the ATF4-CHOP pathway does not fully rely on the phosphorylation of eIF2α although is highly dependent on PP2A activity to trigger cancer cell death.

### PP2A-dependent TFE3 activation of ATF4 and CHOP expression regulates cell fate.

**Figure 5 –:**
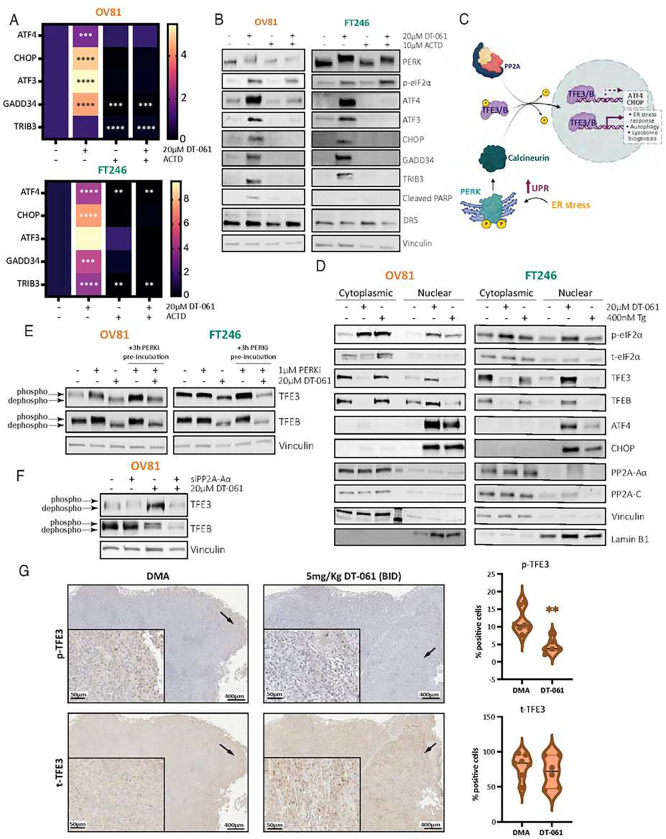
PP2A-dependent activation of ATF4 and CHOP expression is transcriptionally regulated by TFE3. A) mRNA analysis of OV81 and FT246 cell lines assessing transcriptional expression and repression of ATF4, CHOP, ATF3, GADD34, and TRIB3 genes after DT-061 treatment with or without actinomycin D (ActD) pre-incubation, a potent transcription inhibitor that intercalates with the DNA to prevent RNA Polymerase binding. B) Western blotting analysis of the same experimental setup in A) evaluating the dependency of protein expression on transcriptional regulation induced by DT-061 in the OV81 cell line. C) Schematic representing the mechanism by which TFEB and TFE3 are central components of the ER and integrated stress response. TFEB/3 are regulated and activated in the cytoplasm for nuclear transport, where they can act as transcription factors and activate ER stress responses, mTOR-dependent autophagy, and lysosome biogenesis genes. Two different phosphatases have been established to dephosphorylate TFEB/3 in the cytoplasm – Calcineurin (UPR-dependent) and PP2A (UPR-independent) – contingent on the type of stress and cellular context. Schematic designed in biorender. D) Western blotting analysis evaluating cytoplasmic versus nuclear localization and protein expression of TFEB/3, ATF4, and its downstream target genes after 6 hours of DT-061 or Tg treatment in OV81. E) Protein lysates from experiments represented in Fig. 4C were reanalyzed to evaluate TFEB/3 dephosphorylation status and overall expression under PERKi, DT-061, and combination conditions. Western blotting analysis helped evaluate TFEB/3’s dependency on the UPR-dependent regulation via Calcineurin, in the presence of DT-061. F) Protein lysates from Fig. 4F now evaluating TFEB/3 expression and dephosphorylation status upon DT-061 treatment, and thus evaluate dependency profiles of DT-061-mediated TFEB/3 effects on PP2A, the UPR-independent mechanism. G) Representative histology pictures with immunohistochemistry staining (brown) of phospho and total TFE3 for DMA or DT-061 treated tumors from previously published OV81 PDX efficacy studies^21^.

To gain an insight into whether ATF4 and CHOP regulation by DT-061 acts at the transcript level or via alternative mechanisms, we then sought to evaluate the effect of DT-061 on ATF4 mRNA levels. First, we pre-incubated cells for 30 minutes with actinomycin D (ActD), a potent transcription inhibitor, before treatment with DT-061. In both OV81 and FT246 cell lines, ActD inhibits DT-061-stimulated mRNA levels of ATF4 and all its downstream targets (Fig. 5A). Interestingly, co-treatment of ActD with DT-061 also restored the increased expression of ATF4, ATF3, CHOP, GADD34, and TRIB3 proteins back to baseline levels (Fig. 5B), although PERK and p-eIF2α post-translational regulation was efficiently retained as expected. Next, we examined the effect of DT-061 and Tg on the half-life ATF4 and CHOP proteins and observed no significant differences in the ATF4 protein half-life in either OV81 or FT246 treated with DT-061 or Tg (***Supplemental Fig. 3A–3C***). Interestingly, however, CHOP protein half-life (~4.3h) was unaffected by treatment with Tg in OV81 cells and increased by ~2 hours (half-life ~6.0h) upon DT-061-treatment (***Supplemental Fig. 3D*** and ***Supplemental Fig. 3E***). In FT246, CHOP’s half-life increased from 4.8h (DMSO) to 9.7h (DT-061) and 7.3h (Tg), suggesting that, in addition to the considerable effect of DT-061 on mRNA targets of the ATF4 signaling pathway, there may be subtle additional mechanisms that impact on CHOP protein levels.

We next explored the molecular mechanism dictating ATF4 transcriptional upregulation upon DT-061 treatment. Transcription Factor E3 (TFE3) and EB (TFEB) have been recognized as novel and important components of the ISR by directly facilitating transcriptional regulation of ATF4 and CHOP mRNA levels^35^. In addition, these transcription factors have been shown to be activated in the cytoplasm via dephosphorylation by either PP2A^36^ or calcineurin^35,37^, resulting in their nuclear translocation to transcriptionally activate numerous genes (Fig. 5C). To assess whether modulation of PP2A via DT-016 could affect TFE3 and TFEB dephosphorylation and nuclear translocation, we performed nuclear and cytoplasmic fractionation. We found that cellular TFE3 and TFEB were robustly dephosphorylated and translocated into the nucleus upon DT-061 treatment only and not in the presence of 400nM Tg (Fig. 5D). Furthermore, both DT-061 and Tg demonstrated the ability to promote ATF4 and CHOP expression and effectively transport them into the nucleus, however only DT-061 exhibited regulatory control over ATF4 and CHOP transcription through TFE3 and TFEB nuclear shuttling. Next, we examined whether DT-061-triggered effects on TFE3 and TFEB were specifically mediated by PP2A modulation (i.e., independent of calcineurin, a UPR-activated phosphatase). We observed that in the presence of PERKi + DT-061, both TFE3 and TFEB were still dephosphorylated (gel mobility shift assay) (Fig. 5E). Moreover, PP2A-Aα knockdown was sufficient to increase phosphorylated TFE3 (as observed by the shift up in the gel mobility assay, Fig. 5F), but not phosphorylated TFEB. Importantly, DT0–61 was no longer able to induce expression and dephosphorylation of TFE3 in PP2A-Aα knockdown cells, highlighting the activation of TFE3 depends on PP2A activity. The same was not observed for TFEB, as siPP2A exerted little effect on TFEB dephosphorylation to total ratio in the presence or absence of DT-061, although total TFEB protein levels were significantly reduced upon DT-061 treatment. Considering these findings, we examined whether a panel of patient-derived xenograft tumors treated with DT-061 exhibited a change in p-TFE3 localization to determine the relevance of these findings *in vivo*. Immunohistochemistry was performed on tumors treated with 5mg/Kg of DT-061 or control DMA, which we had previously shown to significantly inhibit tumor growth^21^, and stained for phospho and total TFE3 (Fig. 5G). We discovered that p-TFE3 levels decrease markedly in DT-061-treated tumors compared to untreated tumors, while total levels remain unchanged, suggesting that p-TFE3 may serve as a biomarker for the therapeutic efficacy of DT-061.

**Figure 6 –:**
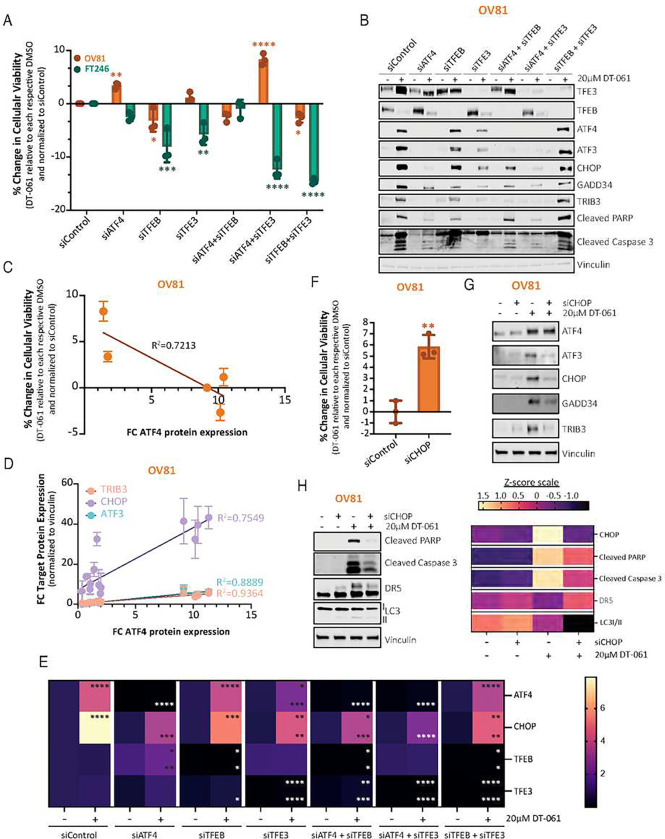
PP2A-mediated TFE3, ATF4, and CHOP activation is required for the DT-061-mediated cytotoxic effects. A) siRNA experiments knocking down ATF4, TFEB, and TFE3 alone or in dual combination (ie., ATF4+TFEB, ATF4+TFE3, and TFEB+TFE3) were performed in OV81 and FT246 to assess the dependency of DT-061-mediated cytotoxic effects on each of these proteins independently or in combination, evaluating recovery profiles in cell viability by cell titer glo analysis. Graphic representation was generated by calculating %change viability relative to siControl DT-061 treated condition for each respective cell line, after normalization to DMSO control. Data are presented as the mean ± SD (n=3), (one-way ANOVA with multiple comparisons, comparing the mean of each column with the mean of siControl DT-061 treated column, *p < 0.05, **p < 0.01, ***p < 0.001, ****p < 0.0001). B), C), D) and E) siRNA conditions utilized in A) were harvested for B) protein and E) RNA analysis. B) Western blotting assessing the effect of each siRNA alone or in dual combination on the molecular changes of TFEB/3, ISR genes (ATF4, ATF3, CHOP, GADD34, and TRIB3), cell death (cleaved PARP, cleaved Caspase 3, and DR5) and autophagy (LC3) markers. C) and D) Correlation analysis graph and *R*^2^ values comparing the expression of ATF4 with C) % change viability or D) each one of its downstream targets (CHOP, TRIB3, and ATF3) in OV81 after DT-061 treatment under RNAi conditions observed in A) and B). E) qPCR analysis evaluating ATF4 and its downstream targets’ genetic expression in OV81 is represented as a bar graph. Data presented as the mean ± SD (n=3) (one-way ANOVA with multiple comparisons, comparing the mean of each column with the mean of siControl DT-061 treated column for each respective target and cell line, ns > 0.05, *p < 0.05, **p < 0.01, ****p < 0.0001, top-relative to siControl DMSO, bottom-relative to its own si DMSO). F) siCHOP cellular viability experiments in OV81 were pursued to assess dependency profiles of DT-061-mediated cytotoxic effects on CHOP protein expression and evaluate recovery in viability measured by cell titer glo analysis. Graph was generated by calculating %change viability relative to siControl DT-061 condition, after normalization to DMSO. Data presented as the mean ± SD (n=3), (one-way ANOVA, *p < 0.05, **p < 0.01, ***p < 0.001, ****p < 0.0001). G) Western blotting analysis in OV81 assessing the effect of siCHOP on the molecular signature downstream of ISR genes (ATF4, ATF3, CHOP, GADD34, and TRIB3), and H) cell death (cleaved PARP, cleaved Caspase 3, and DR5) and autophagy (LC3) markers after treatment with DT-061 (left). Representation by heatmap of Z-score values obtained after western blot quantification using image J and normalization to Vinculin as the loading control (right). Calculated z-scores were obtained from protein quantification of three independent biological replicates.

Next, we sought to determine whether the cytotoxic effects mediated by DT-061 were induced by the PP2A-TFE3-ATF4 pathway. To do so, we performed siRNA experiments, knocking down ATF4, TFEB, and TFE3 alone or in dual combination (i.e., ATF4 + TFEB, ATF4 + TFE3, and TFEB + TFE3). Cell viability data (Fig. 6A) and the expression of death marker proteins (Fig. 6B) indicated that, for OV81 cells, siATF4 or siATF4 + siTFE3 significant contributed to rescuing cellular viability following 9 hours of DT-061 treatment (~3 and ~10% rescue, respectively), with siTFE3 alone also showing a similar trend. These three proteins play crucial roles in maintaining cellular balance and ensuring homeostasis in normal cells treated with DT-061, as any perturbation and combination thereof proved detrimental to viability of FT246 cells (Fig. 6A). OV81 cell viability is associated with reduced ATF4 expression levels (Fig. 6C), which, in turn correlates with decreased expression of all its downstream targets, including ATF3, CHOP, and TRIB3 (Fig. 6D).

Although targeted inhibition of TFEB had no effect on DT-061-mediated induction of ATF4 or CHOP, siRNA knockdown of TFE3 blunted the DT-061-induced upregulation of ATF4 and CHOP by ~2-fold (Fig. 6E). Moreover, the loss of ATF4 and TFE3 together suppressed CHOP activation. Surprisingly, siATF4 at baseline decreased TFE3 levels, suggesting that ATF4 may regulate TFE3 mRNA expression as a negative feedback mechanism, although no previous literature supports this claim. Finally, we examined DT-061-mediated cytotoxicity in cells with siRNA-mediated CHOP knockdown. CHOP knockdown significantly rescued cellular viability in OV81 after 9 hours of DT-061 treatment (Fig. 6F) and reduced the expression of CHOP downstream transcriptional targets (Fig. 6G), while also attenuating the induction of cleaved PARP, cleaved Caspase 3, DR5, and LC3I/II expression (Fig. 6H).

In summary, these findings indicate that DT-061 mediates TFE3 dephosphorylation via PP2A, subsequentially promoting the transcriptional activation of ATF4 and CHOP that drives irreversible ISR phenotypes and triggers pro-death signals in cancer cells.

## DISCUSSION

Targeting PP2A has emerged as an effective therapeutic strategy for the treatment of human cancers due to its unique capability in regulating multiple crucial cellular processes^38^. Intriguingly, despite their multifunctional properties in cellular contexts, PP2A modulators such as DT-061, have demonstrated pre-clinical promise by selectively targeting cancer cells for death while lacking adverse effects on normal tissues^21–28^. Prior research has consistently demonstrated DT-061’s favorable *in vivo* outcomes and tolerability, effectively reducing tumor burden; however, the mechanisms dictating such broad antitumor properties has remained unknown. In the current study, our aim focused on elucidating the intricate mechanisms governing the adaptation of non-transformed cells to chemical PP2A activation, thereby unveiling alternative non-canonical pathways that dictate cellular fate in response to this class of compounds. We have previously demonstrated the high tolerability profiles in non-malignant fallopian tube models to DT-061 *in vitro* after treatment with lower concentrations of DT-061^21^. In the current study, the low-dose colony-forming assays have corroborated the capacity of normal cells to adapt and sustain a durable response, reprograming baseline homeostatic conditions to ensure survival under prolonged stress cues. Intriguingly, both malignant and non-malignant models displayed toxicity phenotypes when exposed to high DT-061 doses (20μM) following 48 hours of consecutive treatment. Subsequent washout studies that recapitulate metabolic clearance revealed that after removing DT-061, non-transformed cells can recover their viability, whereas HGSC cells are irreversibly committed to death. Our research reveals an intrinsic mechanistic vulnerability within cancer cells in the PP2A/TFE3 pathway. DT-061-mediated activation of this pathway triggers an irreversible ISR, resulting in the initiation of pro-death signals governed by ATF4 and CHOP activity, ultimately overpowering adaptive responses. In contrast, non-malignant tissues exhibit ISR plasticity, instigating adaptive homeostatic mechanisms that effectively uphold cellular survival during ISR activation induced by PP2A chemical modulators. This resilience is driven by ATF4 and CHOP-induced transcriptional and translational reprogramming, preventing irreversible ISR, and favoring pro-survival signals.

During cellular stress responses, the ISR rapidly and efficiently resolves cytotoxic and proteolytic stress favoring cell survival and homeostatic recovery mechanisms. However, sustained activation of these pathways results in the exacerbated activation of ATF4, a master regulator of cell fate, thereby accumulating irreversible pro-death signals and determining deleterious cell fates^8,10,39,40^. While PP2A’s role in regulating TFE3 to subsequently transcribe ATF4 and CHOP during ISR has been previously described^36^, its involvement in governing ISR reversibility was never explored. Our studies propose a novel mechanism whereby chemical PP2A modulation chronically activates TFE3, consequently inducing ATF4 and CHOP expression and activity. This ultimately triggers an irreversible cellular stress response in cancer cells, which serves as a novel mechanism for preventing ISR plasticity in this context. We further uncovered that through the dephosphorylation and nuclear translocation of the transcription factor TFE3, DT-061 induces chronic ISR independent of PERK activation. Moreover, our results show that DT-061 modulation of PP2A regulates TFE3 to promote ATF4 expression and activity in both HGSC and non-malignant FT cells, albeit distinctive downstream response mechanisms are activated, resulting in two distinct cell fates. In cancer cells, DT-061 treatment drives specific t-RNA charging signaling pathways to bias the translational machinery toward activating oxidative stress and ISR genes, such as ATF4, CHOP, DR5, and TRIB3, ultimately leading to an increase in autophagy, necroptosis, and apoptosis triggered by chronic and irreversible ISR. On the other hand, non-transformed cells’ priority is to restore normal translational and secretory abilities by shutting down pro-death pathways. Ultimately, our studies unveil that DT-061 initiates two parallel pathways: PERK activation and PP2A-TFE3-mediated induction of the ISR. Importantly, the phosphorylation of eIF2⍺ alone was proved to be insufficient to promote a chronic and irreversible ISR state, requiring TFE3-mediated transcriptional activity to sustain prolonged ATF4 expression and activity.

Recently, selective chemical inhibition of PP2A has been shown to attenuate cellular stress response in plant cells treated with tunicamycin^41^. Nonetheless, the molecular mechanisms underlying PP2A’s regulation these stress mechanisms, and which specific heterotrimeric components are responsible to modulate cellular response to proteolytic and stress imbalances were not identified. Furthermore, the role of PP2A in regulating cellular stress-mediated responses in human cells are lacking, therefore requiring more thorough investigation. To date, literature shows that PP2A forms a complex with RACK1 to dephosphorylate and inactivate IRE1⍺ during ER stress conditions in pancreatic β-cells^42^. Our studies are the first to propose a new role of PP2A in triggering irreversible ISR via TFE3-ATF4 transcriptional and translational expression regulation, selectively targeting cancer cells for death while normal cells adapt. Nonetheless, this manuscript does not fully explore the alternative mechanism dictating ATF4 protein synthesis, and the machinery recruited by PP2A to translate ATF4 mRNA under DT-061 treatment remains elusive. ATF4 mRNA has two upstream open reading frames (uORFs)^43,44^. Under normal conditions, abundant eIF2-t-RNA tertiary complexes (TC) ensure continuous ribosomal translation until encountering the inhibitory uORF2, where translation halts^5,43^. Conversely, during stress, canonical eIF2α phosphorylation reduces eIF2-t-RNA TC efficiency and Met-t-RNA delivery, enabling ribosomes to bypass uORF2. This facilitates the resumption of translation at the mORF, thereby promoting efficient ATF4 protein synthesis. Recently, a distinct mechanism has been proposed to regulate ATF4 mRNA translation independently of the eIF2 complex during ER stress conditions. *Vasudevan et al.* identified the non-canonical initiation factor eIF2D and DENR as essential factors for ATF4 translation during tunicamycin treatment^45^, recruiting Met-t-RNA *de novo* and independently of eIF2⍺^46^. Although approximately 50% of human mRNA rely on leaky scanning and re-initiation processes for mORF recognition, only recently this process has been more carefully molecularly characterized^47–49^. Thus, its role in ATF4 translation in the context of DT-061 is warranted and must be explored in future studies.

Our research further indicates that DT-061 impairs the secretome in both cancer and non-malignant cells. Whereas FT cells recover from ISR by reprogramming overall translation to initiate adaptive homeostasis, HGSC cells irreversibly trigger the ISR and accumulate autolysosomes during DT-061-induced autophagy, leading to cell death. The proteomics studies revealed that DT-061 induces NRF2-mediated oxidative stress in OV81 cells while repressing it in FT246 under the same conditions. Interestingly, NRF-2, a strongly selective gene (DepMap), has been shown to transcriptionally regulate ATF4 levels under oxidative stress conditions^50^, forming a complex with PP2A to induce autophagy, apoptosis, and ROS accumulation in other disease contexts^51^. Given the similarity between these findings and our DT-061 results, further investigation into the NRF-2-PP2A complex formation and activity in DT-061-induced cellular stress and cytotoxicity is warranted.

Our data revealed that DT-061 leads to ATF4-mediated cell death, caused by the chronic accumulation of CHOP-dependent signaling cascades, including apoptosis, autophagy, and ROS. Interestingly, despite previous observation that DT-061 treatment resulted in a G_1_ cell cycle arrest in HGSC^21^; its correlation with cellular stress and CHOP expression were not previously established. CHOP protein upregulation has been formerly shown to mediate G_1_ cell-cycle arrest by interacting with p21, ultimately resulting in apoptosis^52,53^. This suggests CHOP’s possible involvement in the previously observed cell cycle arrest profiles during DT-061 treatment, contributing to its cytotoxic effects in cancer cells. Additionally, DT-061 significantly induced specific ISR stress-mediated apoptotic markers, such as DR5, a downstream transcription target of CHOP^54–56^. Edagawa *et al.* has previously shown that the PERK-ATF4-CHOP pathway mediates DR4 and DR5 activation in an ATF3/CHOP-dependent manner when TP53 is functionally lost or deleted^54^, a characteristic signature of HGS cancers Moreover, DR4/5 induction can also be regulated via the IRE1α/TRAIL arm of the UPR by interacting with phosphorylated C-Jun, forming a complex and regulates DR4/5 transcriptional activity^55^. Studies have shown that the CHOP-DR5 signaling triggers extrinsic apoptosis due to severe and chronic ROS accumulation^57–59^, which also occurs during DT-061 treatment. Previous research has established that CHOP activated by cellular stress induces autolysosomes maturation, contributing to LC3II-dependent autophagy during early stages of stress while inhibiting autophagy-mediated granules that guarantee apoptosis in later stages^60,61^. Such studies suggest CHOP plays a crucial role in the transitioning between autophagy and apoptosis when stress surpasses specific thresholds, possibly explaining distinct outcomes in cancer versus non-transformed cells post DT-061 exposure. Together, our results further validate these findings, confirming that DT-061-mediated PP2A modulation functionally activates these previously established pathways in a CHOP-dependent mechanism.

In summary, our work indicates that DT-061, and potentially other chemical modulators of PP2A, regulate an alternative cellular stress response pathway, activating an irreversible ATF4-CHOP pro-death response in tumor cells but not in normal cells. Beyond HGSC, our research lays a strong foundation for DT-061’s anticancer properties in other malignancies, suggesting this could be a molecular mechanism leveraged by multiple cancer contexts, explaining why DT-061 has such widespread potent activities in a variety of cancers. Therefore, our results indicate that PP2A is a master regulator of ATF4 and CHOP activity during DT-061-mediated ISR and cellular stress responses, highlighting its clinical promise due to its ability to selectively target cancer cells for death while avoiding toxicity effects in normal tissues.

## Materials and Methods

### Protein Digestion and TMT labeling

The samples underwent proteolysis and labeling using the TMT 10-plex method, following the manufacturer’s guidelines (ThermoFisher). After protein reduction with 5mM DTT at 45°C for 30 minutes, cysteine residues were alkylated using 15mM 2-chloroacetamide at room temperature for 30 minutes. Protein precipitation involved adding 6 volumes of ice-cold acetone, followed by an overnight incubation at −20°C. The resulting precipitate was centrifuged, and the pellet air-dried. The dried pellet was resuspended in 0.1M TEAB, and overnight digestion with a trypsin/Lys-C mix (at a 1:25 protease-to-protein ratio, Promega) at 37°C with continuous mixing in a thermomixer was carried out. The TMT 10-plex reagents were dissolved in 41 μl of anhydrous acetonitrile, and labeling was achieved by transferring the entire digest to the TMT reagent vial and incubating at room temperature for 1 hour. The reaction was halted with 8μl of 5% hydroxylamine and further incubated for 15 minutes. Labeled samples were combined and dried using a vacufuge. An offline fractionation of the merged sample (approximately 200μg) into 8 fractions was conducted using a high pH reversed-phase peptide fractionation kit, as per the manufacturer’s instructions (Pierce, 84868). These fractions were then dried and reconstituted in 9μl of a solution containing 0.1% formic acid and 2% acetonitrile in preparation for LC-MS/MS analysis.

### Global proteomics analysis

Raw mass spectrometry files were converted into open mzML format using the msconvert utility of the Proteowizard software suite^62^. MS/MS spectra were searched using the MSFragger database search tool^63^ against an UniProt SwissProt protein sequence database appended with an equal number of decoy sequences^64^. For the analysis of whole proteome data, MS/MS spectra were searched using a precursor-ion mass tolerance of 20 ppm, fragment mass tolerance of 0.6 Da, and allowing C12/C13 isotope errors (0/1/2/3). Cysteine carbamidomethylation (+57.0215) and lysine TMT labeling (+229.1629) were specified as fixed modifications, and methionine oxidation (+15.9949), N-terminal protein acetylation (+42.0106), and TMT labeling of peptide N terminus and serine residues were specified as variable modifications. The search was restricted to fully tryptic peptides, allowing up to two missed cleavage sites. The search results were further processed using the Philosopher toolkit v4.5.1^65^. First, MSFragger output files (in pepXML format) were processed using PeptideProphet^66^ (with the high–mass accuracy binning and semi-parametric mixture modeling options) to compute the posterior probability of correct identification for each peptide to spectrum match (PSM). The resulting pepXML files from PeptideProphet (or PTMProphet) were then processed together to assemble peptides into proteins (protein inference) and to create a combined file (in protXML format) of high-confidence protein groups. Corresponding peptides were assigned to each group. The protein inference file and the individual PSM lists were further processed using the Philosopher filter command as follows. Each peptide was assigned either as a unique peptide to a particular protein group or assigned as a razor peptide to a single protein group that had the most peptide evidence. The protein groups assembled by ProteinProphet^67^ were filtered to 1% protein-level False Discovery Rate (FDR) using the chosen FDR target-decoy strategy and the best peptide approach (allowing both unique and razor peptides) and applying the picked FDR strategy^68^. In each TMT plex, the PSM lists were filtered using a stringent, sequential FDR strategy, retaining only those PSMs with PeptideProphet probability of 0.9 or higher (which in these data corresponded to less than 1% PSM-level FDR) and mapped to proteins that also passed the global 1% protein-level FDR filter. For each PSM that passed these filters, MS1 intensity of the corresponding precursor-ion was extracted using the Philosopher label-free quantification module based on the moFF method^69^ (using 10 p.p.m mass tolerance and 0.4 min retention time window for extracted ion chromatogram peak tracing). In addition, for all PSMs corresponding to a TMT-labeled peptide, TMT reporter ion intensities were extracted from the MS/MS scans (using 0.002 Da window) and the precursor ion purity scores were calculated using the intensity of the sequenced precursor ion and that of other interfering ions observed in MS1 data (within a 0.7 Da isolation window). All supporting information for each PSM, including the accession numbers and names of the protein/gene selected based on the protein inference approach with razor peptide assignment and quantification information (MS1 precursor-ion intensity and the TMT reporter ion intensities) was summarized in the output PSM files, one file for each TMT experiment. The tables were further processed using TMT-Integrator^70^ to generate summary reports at the gene and protein level and, for phosphopeptide enriched data, also at the peptide and modification site levels. In the quantitation step, TMT-Integrator used as input the PSM tables generated by the Philosopher as described above and created integrated reports with quantification across all samples at each level^71^.

### Generation of patient-derived cell lines and cell culture

Patient samples were collected and PD cell lines were generated as previously described^21^. OV81 and CAOV3 cell lines were maintained in DMEM (ThermoFisher Scientific, 10–013-CV), PEO-1 and PEO-C4.2 in RPMI-1640 (Corning, 10–040-CV), and FT246 and FT237 in DMEM-F12 (ThermoFisher Scientific, 10–090-CV), all supplemented with 10% Fetal Bovine Serum (ThermoFisher Scientific, A3382001) and grown at 37 °C in 5% CO_2_. FT237 and FT246 were kindly provided by Dr. Drapkin and PEO cell lines by Dr. Taniguchi.

### Annexin-V and PI staining

Cells were plated overnight and treated the following day with vehicle control (DMSO) or DT-061 for 6 or 24 hours. For each timepoint, media was collected, and cells were washed with cold 1X Phosphate Buffer Saline (PBS) (Fisher Scientific, SH3025601). Cells were then trypsinized, collected, and pelleted by centrifugation at 300g for 5 minutes at 4°C. The supernatant was then aspirated, and the cell pellet was washed once more with cold PBS. Alexa Fluor 488 Conjugate Kit (Invitrogen, A13201) was used to stain cells at room temperature protected from light followed by flow cytometry analysis using a Bio-Rad ZE5 Cell Analyzer.

### LC3-dependent autophagy flux assay

The Premo™ Autophagy tandem sensor RFP-GFP-LC3B kit (ThermoFisher Scientific, P36239) was utilized as a monitoring system of the autophagic flux in OV81 and FT246 cells LC3B-dependent. Cells were seeded in a white-sided with clear bottom 96-well plate (Corning, 3610) overnight with the BacMam 2.0 LC3B-FP reagent, at 37°C in 5% CO_2_. The next day, cells were treated with either DMSO or DT-061 for 3, 6, and 9 hours. The fluorescence signal was acquired using a plate reader.

### Reactive oxygen species accumulation

The MitoRos™ 580 reagent (Fisher Scientific, 50–195-3583) was used to detect live ROS accumulation after 1, 3, 6, and 24 hours of DMSO or DT-061 treatment. OV81 and FT246 cells were seeded in a white-sided with clear bottom 96-well plate overnight at 37°C in 5% CO_2_. The next day, cells were treated with DMSO or DT-061. 30 minutes prior to signal reading, cells were incubated with an equal volume of 2X mitoROS working solution. Hank’s Buffer with 20mM HEPES (HHBS) was used to wash the cells three times and 100μL was added at the final step. The fluorescence signal was obtained using a plate reader and the microscope pictures were taken using an Olympus IX70 fluorescence Microscope.

### Proliferation and colony forming assays

To measure cellular viability, each cell line was seeded in either a 12-well (for 3-(4,5-dimethylthiazol-2-yl)-2,5-diphenyltetrazolium bromide assay (ThermoFisher Scientific, M6494)) or 96-well (for cell titer glo (Promega, G7572)) plate overnight to reach 70% confluency the next day. Cells were then treated with either DMSO (ThermoFisher Scientific, BP231–100), DT-061, Thapsigargin (EMD Millipore, T9033), or PERK inhibitor II (EMD Millipore, GSK-2656157) and incubated for time points respective of each figure.

For colony-forming assays, cells were initially plated at a low density (500 and 100 cells/well for FT246 and OV81, respectively) in biological triplicates in a 6-well plate pre-coated with poly-D-lysine (Fisher Scientific, 08–774-270). After 48 hours, cells were treated with either DMSO, DT-061, or Tg. Drug media was replenished every three days, except for the washout studies for which the media changing properties and frequencies are as described in each respective figure legend. On day 12, cells were fixed and stained using 1% crystal violet solution (Fisher Scientific, C581–25) and Image J software was used to quantify the number of colonies formed.

### Western blotting

Cells were harvested for protein isolation using 1X RIPA Lysis and Extraction Buffer (EMD Millipore, 20–188), 5% glycerol, and a cocktail of phosphatase (Thermo Fisher Scientific, A32957) and protease inhibitors (Thermo Fisher Scientific, A32955). Protein quantification of the extracts was performed using the Pierce BCA Protein Assay kit (Thermo Fisher Scientific, 23227). Samples were then prepared with protein concentrations ranging between 1 and 2μg/μl, 1X LDS buffer (Thermo Fisher, NP0007), 2.5% of 2-Mercaptoethanol (Sigma-Aldritch, M6250–250ML), and 1X RIPA. Samples were then run on a 12% or 4–20% gradient SDS–polyacrylamide electrophoresis gel (BioRad, 4568045 or 4568095) at 70–200V. Proteins were transferred onto a nitrocellulose membrane (Bio-Rad, 1704159) with the quick semi-wet transfer Trans-Blot Turbo machine. Membranes were blocked for 1 hour in 5% non-fat milk (Thermo Fisher Scientific, 50488785) made with 1X Tris-Buffered Saline Tween20 (TBST) buffer (AMRESCO 10791–792). Primary antibodies were incubated in either 3% Bovine Serum Albumin (BSA) (Fisher Scientific, 22070008–3) or 5% milk diluted in 1X TBST and secondaries in 5% milk. Antibodies were purchased from Cell Signaling: BiP (3177), PERK (3192), p-eIF2⍺ Ser51 (3597), t-eIF2⍺ (2103), ATF4 (11815), ATF3 (33593), TRIB3 (43043), DR5 (8074), cleaved Caspase 3 (9661), cleaved PARP (9541), LC3I/II (12741), PP2A-A⍺ (2041), PP2A-C (2041); Santa Cruz: CHOP (sc-166682), Vinculin (sc-73614), GAPDH (sc-47724); Proteintech: GADD34 (10449–1-AP); Sigma-Aldrich: TFE3 (HPA023881); 5) Fortis Life Sciences: TFEB (A700–070); Abcam: Lamin B1 (ab133741), and EMD Millipore: Puromycin (MABE343).

### Gaussia luciferase

Gaussia Luciferase was utilized as a reporter assay to monitor the secretory pathway as previously reported by Badr *et al.*^31^. The plasmid was purchased through Dr. Tannous’ lab by Prolume/NanoLight. OV81 and FT246 cells were seeded on day 1 and treated on day 2 with DMSO, 20μM DT-061, or 5μg/ml of BFA for 3, 6, 12, and 24 hours. Both the media and cell lysates were collected to detect Gaussia luciferase luminescence intensity as indirect measurements of secretion and overall protein expression, respectively. Studies were performed according to the protocol described by Pierce™ Gaussia luciferase Flash Assay Kit (Thermo Fisher Scientific, 16158). Gausia luciferase protein was detected via western blotting (Invitrogen, PIPA1181) and RNA via qPCR analysis using the Gaussia luciferase forward 5’-ATCTGCCTGTCCCACATCAA −3’ and Reverse 5’-GTCCACACACAGATCGACCT-3’ primers.

### SUnSET protein synthesis monitoring

Nascent protein translation was measured utilizing the SUrface SEnsing of Translation (SUnSET), a radioactive free puromycin-based method previously described by Schmidt *et al*.^72^. Pulse-chase experiments were performed as follows: Cells were plated overnight at 37°C in 5% CO_2_. The next day, cells were then treated with either DMSO or DT-061. One hour prior to harvesting, 10 minutes of 10μg/ml puromycin (Sigma-Aldrich, P8833) incubation (pulse) was performed to label nascent polypeptide chains, followed by 50-minute incubation with puromycin-free media containing either DMSO or DT-061 (chase). One wash of cold 1X Phosphate Buffer Saline (PBS) (Fisher Scientific, SH3025601) was used in between the two phases. Cells were then harvested and puromycin labeling was measured via western blotting techniques.

### Quantitative reverse transcriptase polymerase chain reaction analysis

Total RNA was isolated from OV81 and FT246 cells (Norgen Biotek, 48400) for cDNA preparation (Thermo Fisher Scientific, 4387406). cDNA was amplified via PCR using a Roche Lightcycler II real-time PRC machine using gene-specific PCR primers and SYBR Green Master mix (Thermo Fisher Scientific, A46111). 18S was used as a reference gene with the following primer sets: Forward 5’-CATCCTTTACATCCTTCTGTCTGT-3’ and Reverse 5’-GGAAAGCAGACATTGACCTC AC-3’. ATF4: Forward 5’-GCACTTCAAACCTCATGGGTTCTC-3’ and Reverse 5’-GGCTCATACAGATGC CACTATC-3’. ATF3: Forward 5’-GCCATTGGAGAGCTGTCTTC-3’ and Reverse 5’-GGGCCATCT GGAACATAAGA-3’. CHOP: Forward 5’-CAGAACCAGCAGAGGTCACA-3’ and Reverse 5’-AGCT GTGCCACTTTCCTTTC-3’. GADD34: Forward 5’-CTTCTGCCTTGTCTCCAGGA-3’ and Reverse 5’-GAC GCCTCTCCTGAACGATA. TRIB3: Forward 5’-CCGTCTTGGGCCCTATGT-3’ and Reverse 5’-GT ACCAGCCAGGACCTCAGT-3’. TFEB: Forward 5’-CCTGGAGATGACCAACAAGCAG-3’ and Reverse 5’-TAGGCAGCTCCTGCTTCACCAC-3’. TFE3: Forward 5’-GATCATCAGCCTGGAGTCCAGT −3’ and Reverse 5’-AGCAGATTCCCTGACA CAGGCA-3’.

### Small interference RNA

siRNA molecules targeting human ATF4 (s1704), CHOP (s3997), TFE3 (s14030), and TFEB (s15495) were purchased from Thermo Fisher Scientific and diluted in the provided nuclease-free molecular grade water. Silencer Select Negative control (4390843) was used as the appropriate control. MISSION esiRNA targeting human PPP2R1A (EHU071351) was obtained from Sigma-Aldrich and diluted nuclease-free TE buffer (10 mM Tris-HCl, pH 8.0, 1 mM EDTA). Lipofectamine RNAiMAX (Thermo Fisher Scientific, 13778150) was used to perform all transfections.

### Nuclear and cytoplasmic protein extraction

NE-PER nuclear and cytoplasmic protein isolation was performed as recommended by the Thermo Scientific kit (78835). Each reagent was supplemented with a phosphatase (Thermo Fisher Scientific, A32957) and a protease inhibitor (Thermo Fisher Scientific, A32955) tablet for a complete extraction buffer preparation.

### Statistical methods

Statistical methods used in the current manuscript are described in detail in each respective figure legend. Fold change calculations, appropriate controls, and normalization approaches are further specified. All quantifications were statistically evaluated using PRISM software, except for global proteomics and RNA-sequencing.
